# Cytogeography and chromosomal variation of the endemic East Asian herb *Lycoris radiata*

**DOI:** 10.1002/ece3.5252

**Published:** 2019-05-22

**Authors:** Kun Liu, Weiqi Meng, Lu Zheng, Lida Wang, Shoubiao Zhou

**Affiliations:** ^1^ Anhui Provincial Key Laboratory of the Conservation and Exploitation of Biological Resources College of Life Sciences Anhui Normal University Wuhu China; ^2^ Anhui Provincial Engineering Laboratory of Water and Soil Pollution Control and Remediation College of Environmental Science and Engineering Anhui Normal University Wuhu China

**Keywords:** diploid, geographic distribution, intraspecific variation, karyotype, *Lycoris radiata*, polyploidy

## Abstract

Information on the spatial distribution of cytotypes and karyotype variation in plants is critical for studies of the origin and evolution of polyploid complexes. Here, the spatial distribution of cytological races and intraspecific variation in the karyotype of *Lycoris radiata*, an endemic species to East Asia, is investigated. Conventional karyotype analysis methods were used to determine ploidy level and karyotypical characteristics in 2,420 individuals from 114 populations of *L*. *radiata* nearly covering the whole distribution areas in China. Of 114 populations studied, 52 (45.61%), 58 (50.88%), and 4 (3.51%) are diploid, triploid, and mixoploid populations, respectively, with 1,224, 1,195, and 1 individuals being diploid, triploid, and tetraploid, respectively. The triploid possesses a much wider distribution range than the diploid, with the former almost occupying the entire range of this complex species in East Asia and the latter distributing in the middle and east regions of China. Triploids tend to occur at high altitudes, and the relationship between the ploidy and altitude is significantly positive but low (*r* ^2 ^= 0.103, *p* < 0.01). About 98.6% of examined bulbs have a common karyotype consisting of 22 or 33 acrocentric (*A*) chromosomes. Some aberrant chromosomes which should be generated from A‐type chromosome have been found including metacentrics (*m*), small metacentrics (*m*′), and B‐type chromosome. The results can provide a fundamental cytogeographic data for further studies on the evolutionary origins and adaptive divergences of polyploids, especially the triploid, within *L*. *radiata* using molecular and/or ecological methods in the future.

## 1 INTRODUCTION

*Lycoris radiata* (L'Hérit.) Herb. (Amaryllidaceae) is a perennial, bulbous plant distributed from southwestern China eastward to Japan and South Korea, and is one of the most widely distributed species in genus *Lycoris* (Hsu, Kurita, Yu, & Lin, 1994). Since 1920s when the chromosome number of this taxon was first counted (Nishiyama, 1928), much work has been done on the chromosome number and karyotype of this species, particularly on the Japanese islands and East China (Bose, 1963; Kurita, 1987; Liu, Zheng, Xia, & Zhou, 2016; Qin, Zhou, & Wang, 2004; Shao, Yang, Zhang, & Nie, 1994; Zhou et al., 2004; Zhou, Yu, Luo, Hu, & Bi, 2007). For a long time, this species had been thought to harbor only two ploidy levels, diploid (2*n* = 22) and triploid (2*n* = 33), until about a decade ago the tetraploid was firstly found (Zhou et al., 2007). This species exhibits great variation in karyotypes and chromosome number throughout its geographical range. The main chromosome numbers observed in this species complex are as follows: 2*n* = 21, 22, 32, 33, and 44 corresponding to the abnormal diploid, diploid, abnormal triploid, triploid, and tetraploid levels, respectively (Liu et al., 2016; Shi, Qiu, Li, Wu, & Fu, 2006). Cytogeographic patterns on the Japanese islands and South Korea have been depicted clearly, based on 58 and 11 populations of *L. radiata*, respectively, and only triploids were discovered in Japan and South Korea (Chung, 1999; Kurita, 1987). In China, diploid, triploid, and tetraploid plants have been found (Liu et al., 2016; Zhou et al., 2007). However, compared with researches on the populations in Japan, cytological investigations of the Chinese populations, especially those populations in southwest and southeast of China, are cursory to some extent.

As part of a broader investigation into the chromosomal variation and evolution of *Lycoris* species, our objective in this study was to examine the diversity and distribution of *L. radiata* cytotypes in China. Particularly, we addressed the following questions: (a) what are the frequencies of diploid, triploid, and tetraploid plants? (b) what are the geographical patterns of cytotype variation? (c) do polyploids have a wider distribution range than diploid?

## 2 MATERIALS AND METHODS

### 2.1 Plant materials

*Lycoris radiata* is mainly distributed in middle east, southwest, and southeast regions of China, preferring such place as riversides and the edges of farmlands, or growing under evergreen or deciduous broad‐leaved forests. Two thousand four hundred and twenty individuals of *L. radiata* were collected from 114 populations during the past decade (from 2007 to 2017) nearly throughout its distribution range (Table 1). Bulbs collected were more than 3 m apart to avoid biasing sampling of the same clone due to extensive vegetative propagation in this species. All bulbs collected from the wild were cultivated in experiment garden. The corresponding voucher specimens were deposited in Anhui Normal University.

**Table 1:** Origins of materials and the karyotypes

Pop.	Localities (voucher)	Coordinates	Altitude (*m*)	Number of bulbs examined	Chromosome formula	Ploidy
1	Laojiahe Village, Lu'an, Anhui; LR16001	31°29′N/115°24′E	455	22	2*n* = 22A	2*x*
2	Maotanchang Town, Lu'an, Anhui; LR16021	31°19′N/116°32′E	164	20	2*n* = 22A	2*x*
3	Hengdu Town, Chizhou, Anhui; LR16035	30°11′N/117°33′E	78	40	2*n* = 22A	2*x*
4	Bagongshan, Huainan, Anhui; 90409	32°37′N/116°47′E	103	20	2*n* = 22A	2*x*
5	Marenshan, Wuhu, Anhui; LR15002	30°58′N/118°09′E	150	18	2*n* = 22A	2*x*
6	Fenghuangshan, Tongling, Anhui; LR15001	30°51′N/118°01′E	180	30	2*n* = 22A	2*x*
7	Dashan Village, Chizhou, Anhui; LR14002	30°01′N/117°21′E	340	25	2*n* = 22A	2*x*
8	Qinyunshan, Huangshan, Anhui; LR13016	29°48′N/118°02′E	265	24	2*n* = 22A	2*x*
9	Wenquan Town, Anqing, Anhui; LR15003	30°56′N/116°17′E	628	6	2*n* = 22A	2*x*
10	Eshan Town, Wuhu, Anhui; 708251	31°06′N/118°17′E	95	14	2*n* = 22A	2*x*
11	Yashan, Wuhu, Anhui; LR12001	30°48′N/117°59′E	310	21	2*n* = 22A	2*x*
12	Nanquan Village, Tongling, Anhui; LR13003	30°25′N/117°16′E	38	30	2*n* = 22A	2*x*
13	Xiaotian Town, Lu'an, Anhui; LR13009	31°11′N/116°35′E	138	13	2*n* = 22A	2*x*
14	Daguling, Yi County, Huangshan, Anhui; LR11002	30°04′N/117°48′E	281	10	2*n* = 22A	2*x*
15	Xin'anjiang, Huangshan, Anhui; LR11003	29°39′N/118°11′E	132	5	2*n* = 22A	2*x*
16	Shitan Town, Qingyuan, Guangdong; LR16046	24°11′N/112°40′E	130	26	2*n* = 22A	2*x*
17	Fanjingshan Village, Tongren, Guizhou; LR15010	27°50′N/108°48′E	642	22	2*n* = 22A	2*x*
18	Wuchenhe Town, Xinyang, Henan; LR16034	31°45′N/114°47′E	66	41	2*n* = 22A	2*x*
19	Wufeng Town, Yichang, Hubei; LR16002	30°11′N/110°41′E	760	11	2*n* = 22A	2*x*
20	Rongmei Town, Enshi, Hubei; LR16028	29°49′N/109°54′E	929	15	2*n* = 22A	2*x*
21	Shadaogou Town, Enshi, Hubei; LR16030	29°41′N/109°36′E	628	22	2*n* = 22A	2*x*
22	Jiangyangping Town, Yichang, Hubei; LR16032	31°06′N/110°50′E	543	20	2*n* = 22A	2*x*
23	Beifeng Village, Yichang, Hubei; LR16007	31°06′N/110°49′E	544	40	2*n* = 22A	2*x*
24	Shaping Town, Xianning, Hubei; LR15005	29°22′N/113°50′E	128	26	2*n* = 22A	2*x*
25	Wudangshan, Shiyan, Hubei; LR16040	32°26′N/111°03′E	353	11	2*n* = 22A	2*x*
26	Maogou Town, Xiangxi, Hunan; LR15030	28°35′N/109°22′E	273	30	2*n* = 22A	2*x*
27	Miaoshi Town, Changde, Hunan; LR16018	29°28′N/111°12′E	145	10	2*n* = 22A	2*x*
28	Wulingyuan, Zhangjiajie, Hunan; LR15016	29°21′N/110°29′E	423	28	2*n* = 22A	2*x*
29	Biyunfeng, Yiyang, Hunan; LR15017	28°26′N/112°22′E	488	23	2*n* = 22A	2*x*
30	Hougushan, Chenzhou, Hunan; LR15020	25°52′N/113°17′E	150	26	2*n* = 22A	2*x*
31	Lujiawan Village, Huaihua, Hunan; LR16022	28°35′N/110°27′E	123	20	2*n* = 22A	2*x*
32	Jiemuxi Town, Huaihua, Hunan; LR16024	28°47′N/110°25′E	236	20	2*n* = 22A	2*x*
33	Daping Town, Zhangjiajie, Hunan; LR16026	29°33′N/110°04′E	502	40	2*n* = 22A	2*x*
34	Liangshuikou Town, Zhangjiajie, Hunan; LR16027	29°00′N/110°30′E	349	43	2*n* = 22A	2*x*
35	Yixing forest park, Wuxi, Jiangsu; LR13001	31°17′N/119°45′E	70	23	2*n* = 22A	2*x*
36	Shipai Town, Suzhou, Jiangsu; LR11004	31°33′N/121°01′E	9	16	2*n* = 22A	2*x*
37	Zhuangkou Town, Ganzhou, Jiangxi; LR15033	25°40′N/115°39′E	160	23	2*n* = 22A	2*x*
38	Yangxi Town, Ji'an, Jiangxi; LR15035	27°18′N/114°12′E	196	8	2*n* = 22A	2*x*
39	Tongtianyan, Ganzhou, Jiangxi; LR15022	25°55′N/114°54′E	134	28	2*n* = 22A; 2*n* = 20A+1m	2*x*
40	Nashan Village, Jinggangshan, Jiangxi; LR15023	26°43′N/114°16′E	238	30	2*n* = 22A	2*x*
41	Longhushan, Yingtan, Jiangxi; LR15025	28°05′N/116°58′E	120	28	2*n* = 22A	2*x*
42	Sishiba Town, Shangrao, Jiangxi; LR15026	28°12′N/118°02′E	169	25	2*n* = 22A	2*x*
43	Beishan, Jinhua, Zhejiang; LR15027	29°12′N/119°37′E	501	33	2*n* = 22A	2*x*
44	Yantou Town, Wenzhou, Zhejiang; LR15028	28°20′N/120°43′E	53	31	2*n* = 22A	2*x*
45	Chichengshan, Taizhou, Zhejiang; LR15029	29°10′N/121°01′E	240	33	2*n* = 22A	2*x*
46	Jiufengshan, Zhejiang; LR13012	29°00′N/119°22′E	601	16	2*n* = 22A	2*x*
47	Jingling Town, Shaoxing, Zhejiang; LR13014	29°22′N/120°47′E	238	18	2*n* = 22A	2*x*
48	Yangduan Village, Jiujiang, Jiangxi; LR15004	29°32′N/115°22′E	510	24	2*n* = 22A	2*x*
49	Longpan Town, Nanchong, Sichuan; LR17001	30°49′N/105°53′E	386	10	2*n* = 22A	2*x*
50	Dayang Town, Lishui, Zhejiang; LR15032	28°31′N/120°11′E	900	18	2*n* = 22A	2*x*
51	Xidian Town, Ningbo, Zhejiang; LR15037	29°26′N/121°25′E	35	6	2*n* = 22A	2*x*
52	Qingliangfeng Town, Hangzhou, Zhejiang; LR13013	30°06′N/118°54′E	222	19	2*n* = 22A	2*x*
53	Jingtingshan, Xuancheng, Anhui; LR11001	30°59′N/118°43′E	161	25	2*n* = 33A	3*x*
54	Langyashan, Chuzhou, Anhui; LR08002	32°16′N/118°16′E	211	36	2*n* = 33A	3*x*
55	Huangpushan, Chuzhou, Anhui; 803271	32°20′N/118°00′E	151	30	2*n* = 33A	3*x*
56	Sanqi Town, Lu'an, Anhui; LR13010	31°14′N/116°41′E	176	8	2*n* = 33A	3*x*
57	Meijie Town, Chizhou, Anhui; LR12003	30°27′N/117°34′E	120	18	2*n* = 33A	3*x*
58	Longshe Town, Pengshui, Chongqin; LR16005	27°07′N/108°11′E	932	6	2*n* = 33A	3*x*
59	Xiannvshan Town, Wulong, Chongqin; LR16006	29°24′N/107°47′E	812	36	2*n* = 33A	3*x*
60	Wenfeng Town, Wuxi, Chongqing; LR16031	31°25′N/109°10′E	1,061	1	2*n* = 33A	3*x*
61	Jinhan Town, Ningde, Fujian; LR16042	26°41′N/119°28′E	148	26	2*n* = 33A	3*x*
62	Maixieyan Village, Putian, Fujian; LR16043	25°32′N/118°48′E	654	15	2*n* = 31A + 1m + 1B	3*x*
63	Heping Town, Zhangzhou, Fujian; LR16044	23°56′N/117°10′E	665	26	2*n* = 33A	3*x*
64	Laizhou Town, Nanping, Fujian; LR08006	26°37′N/117°58′E	450	10	2*n* = 33A	3*x*
65	Tianbaoyuan Reserve, Yong'an, Fujian; LR08004	25°58′N/117°22′E	715	15	2*n* = 33A	3*x*
66	Heshui Village, Shaoguan, Guangdong; LR16019	24°53′N/113°56′E	240	30	2*n* = 33A	3*x*
67	Wangbian Village, Shaoguan, Guangdong; LR15021	25°04′N/113°19′E	130	21	2*n* = 33A	3*x*
68	Lvtian Town, Conghua, Guangdong; LR16045	23°48′N/113°55′E	224	30	2*n* = 33A	3*x*
69	Licheng Town, Guilin, Guangxi; LR15006	24°29′N/110°24′E	148	24	2*n* = 33A	3*x*
70	Rongjiang Town, Guilin, Guangxi; LR15007	25°41′N/110°19′E	293	39	2*n* = 33A	3*x*
71	Tangjia Village, Guilin, Guangxi; LR15008	25°19′N/110°19′E	156	5	2*n* = 31A+1m + 1B	3*x*
72	Pingxi Village, Qindongnan, Guizhou; LR16014	27°07′N/107°46′E	716	30	2*n* = 33A	3*x*
73	Zhaibao Village, Tongren, Guizhou; LR15011	27°46′N/108°45′E	436	11	2*n* = 33A	3*x*
74	Dongfeng Town, Guiyang, Guizhou; LR15012	26°38′N/106°49′E	1,015	5	2*n* = 33A	3*x*
75	Ali Village, Guiyang, Guizhou; LR15013	26°34′N/106°48′E	1,052	25	2*n* = 33A	3*x*
76	Chengguan Town, Pan County, Liupanshui, Guizhou; LR15014	25°47′N/104°40′E	1,625	8	2*n* = 31A + 1m + 1m′	3*x*
77	Yaojiatun Village, Anshun, Guizhou; LR15015	26°12′N/105°54′E	1,399	27	2*n* = 33A	3*x*
78	Hongshan Town, Suizhou, Hubei; LR15034	31°36′N/112°55′E	210	21	2*n* = 33A	3*x*
79	Cihe Town, Xiangyang, Hubei; LR15036	32°01′N/111°48′E	166	24	2*n* = 33A	3*x*
80	Bajiaodongzu Town, Enshi, Hubei; LR16004	30°07′N/109°23′E	590	24	2*n* = 33A	3*x*
81	Moudao Town, Lichuan, Hubei; LR16020	30°29′N/108°39′E	1,032	25	2*n* = 33A	3*x*
82	Zhonglu Town, Enshi, Hubei; LR16041	30°08′N/108°45′E	1,088	31	2*n* = 33A	3*x*
83	Majiawan, Hefeng County, Enshi, Hubei; LR16029	29°52′N/110°00′E	583	1	2*n* = 33A	3*x*
84	Xiaojiacun Town, Yongzhou, Hunan; LR16015	26°21′N/112°00′E	103	20	2*n* = 33A	3*x*
85	Dankou Town, Shaoyang, Hunan; LR16013	26°25′N/110°13′E	400	25	2*n* = 33A	3*x*
86	Bozhou Town, Huaihua, Hunan; LR16017	27°23′N/109°17′E	325	24	2*n* = 33A	3*x*
87	Yidushui Town, Shaoyang, Hunan; LR15009	26°34′N/111°12′E	349	29	2*n* = 33A	3*x*
88	Qingwei Village, Loudi, Hunan; LR15018	27°51′N/110°58′E	626	12	2*n* = 33A	3*x*
89	Liuxin Village, Loudi, Hunan; LR15019	27°51′N/110°59′E	693	31	2*n* = 33A	3*x*
90	Longtan Village, Zhuzhou, Hunan; LR16008	26°09′N/113°45′E	480	20	2*n* = 33A	3*x*
91	Huaguoshan, Lianyungang, Jiangsu; LR13002	34°39′N/119°16′E	131	15	2*n* = 33A	3*x*
92	Laoshan, Nanjing, Jiangsu; LR14001	32°06′N/118°36′E	104	3	2*n* = 33A	3*x*
93	Yushan, Lianyungang, Jiangsu; LR13004	34°38′N/119°15′E	30.9	13	2*n* = 33A	3*x*
94	Wutong Town, Guilin, Guangxi; LR16016	25°22′N/110°03′E	180	28	2*n* = 33A	3*x*
95	Wanger Town, Shangrao, Jiangxi; LR15031	28°17′N/117°30′E	66	22	2*n* = 33A	3*x*
96	Chenshan, Shanghai; LR16010	31°04′N/121°10′E	25	38	2*n* = 33A	3*x*
97	Sheshan, Shanghai; LR16011	31°05′N/121°11′E	57	37	2*n* = 33A	3*x*
98	Longwangou, Lueyang County, Shannxi; LR13015	33°22′N/106°09′E	692	6	2*n* = 33A	3*x*
99	Liyushan, Ankang, Shannxi; LR16036	32°41′N/108°55′E	300	6	2*n* = 33A	3*x*
100	Taibai Village, Hanzhong, Shannxi; LR16039	33°00′N/106°47′E	554	36	2*n* = 33A	3*x*
101	Dahanshan, Hanzhong, Shannxi; LR16038	32°57′N/106°56′E	950	6	2*n* = 33A	3*x*
102	Taibaishan, Baoji, Shannxi; LR16039	34°05′N/107°42′E	1,136	6	2*n* = 33A	3*x*
103	Motan Town, Guangyuan, Sichuan; LR16003	32°10′N/106°03′E	868	24	2*n* = 33A	3*x*
104	Dafo Town, Leshan, Sichuan; LR10001	29°46′N/104°03′E	436	27	2*n* = 33A	3*x*
105	Changyuangou Village, Nanchong, Sichuan; LR16009	30°51′N/105°59′E	310	15	2*n* = 33A	3*x*
106	Dayun Village, Yibin, Sichuan; LR13005	28°22′N/104°46′E	597	30	2*n* = 33A	3*x*
107	Futou Village, Yibin, Sichuan; LR13006	28°20′N/104°53′E	447	31	2*n* = 33A	3*x*
108	Gaojian Village, Yibin, Sichuan; LR13007	28°17′N/104°59′E	604	30	2*n* = 33A	3*x*
109	Emeishan, Leshan, Sichuan; LR16033	29°36′N/103°23′E	880	6	2*n* = 33A	3*x*
110	Baishanzu Town, Lishui, Zhejiang; LR08005	27°43′N/119°12′E	1,074	5	2*n* = 33A	3*x*
111	Huilongchang Village, Nanchong, Sichuan; LR16012	30°53′N/105°59′E	387	6	2*n* = 22A; 2*n* = 33A	2*x*(1) + 3*x*(5)
112	Lianhua Village, Wuhu, Anhui; LR13011	31°03′N/117°32′E	223	13	2*n* = 22A; 2*n* = 33A	2*x*(11) + 3*x*(2)
113	Caishiji park, Ma'anshan, Anhui; LR08003	31°39′N/118°27′E	101	26	2*n* = 22A; 2*n* = 22A + 1B; 2*n* = 33A; 2*n* = 31A + 1m + 1m′	2*x*(16) + 3*x*(10)
114	Tangxi Town, Chizhou, Anhui; LR12002	30°20′N/117°36′E	112	36	2*n* = 22A; 2*n* = 44A	2*x*(35) + 4*x*(1)

**Figure 1:**
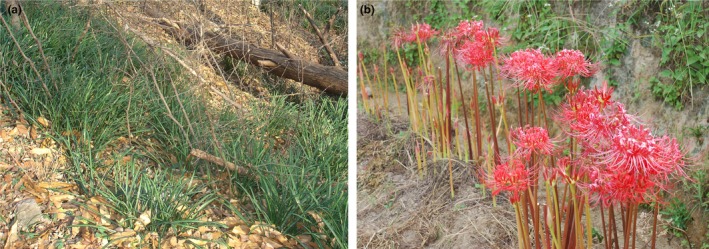
Photograph of *Lycoris radiata*. (a) Plants in vegetative phase in habitat. (b) Plants in flowering in habitat

The growth form and seasonality of *L. radiata* are very characteristic, that is, the productive and reproductive phases are separate (Figure 1a,b). The diploids can produce seeds and have sexual and asexual reproduction, while the triploids can only propagate by clone, producing no seed.

### 2.2 Karyotype analysis

All bulbs with the old roots cut were maintained in wet soil/tap water culture, and conventional karyotype analysis methods (Zhou et al., 2007) were used. The karyotype formula was based on the measurements of mitosis metaphase chromosomes taken from two or three well‐spread metaphase cells. For the karyotype description and comparison, the simplified symbols were adapted according to Levan, Fredga, and Sandberg (1964), Kurita (1986), and Liu et al. (2012): *m* for large metacentric chromosome with arm ratio of 1.00–1.70; *m*′ for small metacentrics; *st* for subtelocentric chromosome with arm ratio of 3.01–7.00; *t* for acrocentric chromosome with arm ratio of 7.01–20.0; *T* for telocentric chromosome having mostly terminal centromere with dot‐like short arm whose length is very short and with the arm ratio being more than 20.0; A‐type chromosome includes both st‐ and t‐type chromosome; SAT for A‐type satellite chromosome; *B* for very small chromosome; *r* means arm ratio.

### 2.3 Analysis of cytotype distribution

The relationship between altitude and ploidy of the sampled populations was tested using Pearson correlation analysis by SPSS v22.0. In order to exactly reveal the geographical distribution patterns of each cytotype of *L. radiata* complex in East Asia, we choose 43 previously published populations with precise chromosome number data and geographical location or longitude and latitude information, of which 7 populations (Liu et al., 2016), 29 populations (Kurita, 1987), and 7 populations (Chung, 1999) were from China, Japan, and South Korea, respectively. In total, 157 populations with exact ploidy data were mapped using ArcMap 10.0.

## 3 RESULTS

### 3.1 The ploidy and chromosome number of *Lycoris radiata*

A total of 2,447 individuals, from 114 populations of *Lycoris radiata* (Table 1), were examined to determine the chromosome number and karyotype. Of 114 populations investigated, 52 (45.61%), 58 (50.88%), and 4 (3.51%) were diploid, triploid, and mixoploid populations, respectively. A total of 1,224 bulbs and 1,195 bulbs are diploid and triploid, respectively, with a few bulbs possessing abnormal chromosomes and B chromosomes, and only one bulb from population 114 is tetraploid having 2*n* = 4*x* = 44 (Table 1).

### 3.2 Karyomorphology

**Figure 2:**
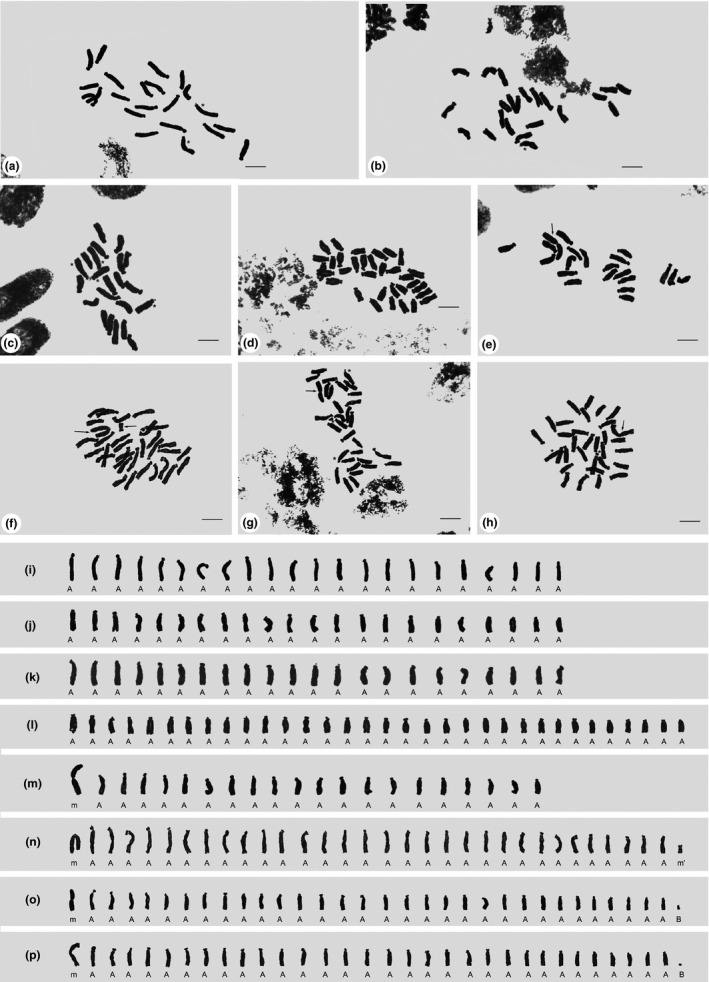
The metaphase chromosome morphology and karyotypes. (a, i) a metaphase chromosome from population 114. Asterisks indicate two SAT chromosomes. (b, j) a metaphase chromosome from population 4. Asterisk indicates one SAT chromosomes. (c, k) a metaphase chromosome from population 114. Asterisks indicate four SAT chromosomes. (d, l) a metaphase chromosome from population 77 showing a representative karyotype of triploid. (e, m) a metaphase chromosome from population 39. The arrow indicates one m‐type chromosome. (f, n) a metaphase chromosome from population 76. Arrow indicate one m‐type chromosome and one *m*′‐type chromosome. (g, o) a metaphase chromosome from population 71. The arrow indicates one m‐type chromosome. Asterisks indicate two SAT chromosomes and one B‐type chromosome. (h, p) a metaphase chromosome from population 62. The arrow indicates one m‐type chromosome. Asterisks indicate one SAT chromosomes and one B‐type chromosome

The karyotypes of 1,221 bulbs are all composed of 22 A‐type chromosomes, of which 0–4 are satellite chromosomes. One example of representative chromosome constitution of diploid bulb from population 114 at Tangxi Zhen of Chizhou city is shown in Figure 2a,i. The representative karyotype consists of twenty‐two A‐type chromosomes of which two are SAT chromosomes. The measured and calculated values of each chromosome of the representative karyotype of diploid are summarized in Table 2. The short‐arm length of both SAT chromosomes is much the same, and their *r* value is 10.73 and 9.27, respectively. Different types of SAT chromosome combination are observed in the inter‐ and intra‐population. One SAT chromosome is observed in one bulb from population 4 in Bagongshan of Huainan city (Figure 2b,j). As shown in Figure 2c,k four SAT chromosomes are found in one bulb from population 114. The four SATs, reported for the first time here, are the maximum number of SAT chromosome in this species known so far. Of 1,195 triploid bulbs, 1,166 bulbs are typical triploid consisting of 33 A‐type chromosomes with 0–3 SAT chromosomes. A representative karyotype of triploid is shown in Figure 2d,l.

**Table 2:** Measurements of somatic chromosomes in a representative karyotype of diploid

No.	Relative length			Arm ratio	Type
	SL	LL	TL		
1	0.83	4.61	5.44	5.57	A
2	0.75	4.41	5.16	5.89	A
3	0.59	4.57	5.16	7.73	A
4	0.71	4.41	5.12	6.22	A
5	0.43	4.65	5.08	10.73	A*
6	0.47	4.41	4.89	9.33	A
7	0.55	4.33	4.89	7.86	A
8	0.67	4.14	4.81	6.18	A
9	0.51	4.06	4.57	7.92	A
10	0.59	3.98	4.57	6.73	A
11	0.75	3.78	4.53	5.05	A
12	0.63	3.86	4.49	6.13	A
13	0.51	3.94	4.45	7.69	A
14	0.43	4.02	4.45	9.27	A*
15	0.51	3.90	4.41	7.62	A
16	0.63	3.74	4.37	5.94	A
17	0.67	3.55	4.22	5.29	A
18	0.32	3.66	3.98	11.63	A
19	0.59	3.27	3.86	5.53	A
20	0.55	3.31	3.86	6.00	A
21	0.32	3.55	3.86	11.25	A
22	0.63	3.19	3.82	5.06	A

Note

Asterisk indicates SAT chromosome.

Abbreviations: LL, relative length of long arm; SL, relative length of short arm; TL, total relative length; SL + LL=TL.

Some aberrant chromosomes which should be generated from A‐type chromosome are found including metacentrics (*m*), small metacentrics (*m*′), and B‐type chromosome (Table 3). The occurrence of Robertsonian fusion of A‐type chromosomes is confirmed in some bulbs from five populations, for example, population 39, 62, 71, 76, and 113 26, 32, 40, 60, and 72. The karyotype of one bulb from population 39 in Tongtianyan mountain of Ganzhou city comprises two types of chromosome (A‐ and m‐type chromosome), formulated as 2*n* = 21 = 20*A* + 1*m* (Figure 2e,m). Eight bulbs from population 76 in Pan County and one bulb from population 113 at Caishiji park are abnormal triploids and have 33 chromosomes with 31 A‐type chromosomes, one metacentric chromosome (m‐type chromosome) and a small metacentric chromosome (*m*′‐type chromosome). The karyotype of them is formulated as 2*n* = 33 = 31*A* + 1*m* + 1*m*′ (Figure 2f,n). In addition, five bulbs from population 71 at Tangjia village of Guilin city and fifteen bulbs from population 62 at Maixie village of Putian city also have the m‐type chromosome, and the karyotype of them is formulated as 2*n* = 33 = 31*A* + 1*m* + 1*B* (Figure 2g,h,o,p). Two and one SAT chromosomes are observed in the population 71 and 62, respectively. The arm ratio of m‐type chromosome in the bulbs from population 71 and population 62 is 1.0 and 1.1, respectively. Small B‐type chromosome is also observed in two bulbs from population 113, with the karyotype formulated as 2*n* = 22 = 22*A* + 1*B*. Both *m*′‐type chromosome and B‐type chromosome should originate from A‐type chromosomes, but the former has at least twofold length than the latter.

**Table 3:** The percentage of bulbs having aberrant chromosomes

Ploidy level	Number of bulbs examined	Number of bulbs with aberrant chromosomes		
		*m*	*m*′	*B*
Diploid	1,224	1	0	2
Triploid	1,195	20	9	20
Tetraploid	1	0	0	0
Total number of bulbs	2,420	21	9	22

### 3.3 Distribution of different cytotypes in China

**Figure 3:**
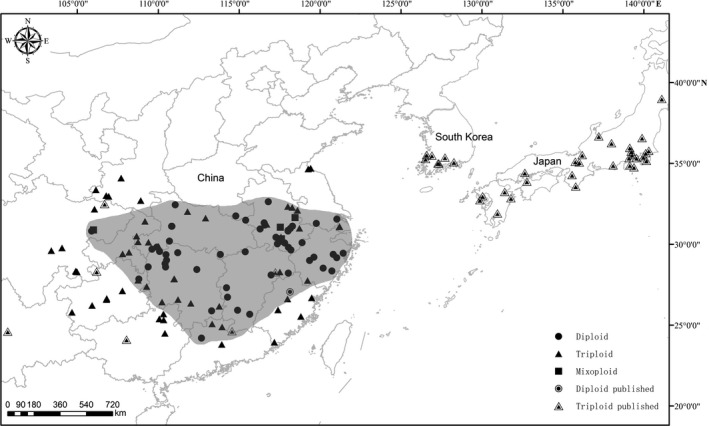
Distribution map of cytologically investigated and previously published populations of *Lycoris radiata* in East Asia. Shaded areas show the distribution range of diploid to our knowledge

Diploid cytotypes are located primarily in the middle and east regions of China. Isolated diploid populations are found in southeast areas of China. The triploid cytotypes have a wider distribution, occupying nearly the whole distribution range of this species in China. Six provinces located on the periphery of the distribution area have no more than one diploid population (Figure 3).

**Figure 4:**
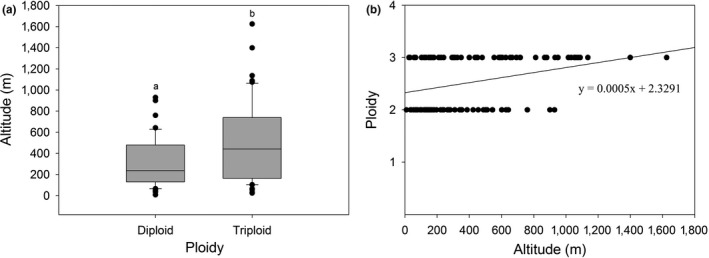
(a) Altitudes comparing ploidy levels. Means significantly different at *p* < 0.01 are indicated by the different letter (two‐tailed unpaired *t* test). Horizontal lines represent the median, and boxes, and whiskers, respectively, the interquartile range and the nonoutlier ranges. Black circles denote outliers. (b) Scatter plot of ploidy (diploid and triploid) versus altitude (meters). The linear relationship shows the significantly positive association between the ploidy and altitude (*r* ^2 ^= 0.103, *p* < 0.01)

In Figure 4, it is indicated that the triploids have a broader altitudinal range than the diploids. The triploids grow over a broad range of altitudes, from 25 to 1,625 m, with the average being 513.39 ± 382.53 m, while the diploids have a narrower altitudinal range of 9–929 m, with an average of 299.06 ± 226.76 m. The difference in mean altitude of localities between diploid and triploid is highly significant (*p* < 0.01; Figure 4a). Moreover, a significantly positive but low correlation was found between the ploidy and altitude (*r* ^2 ^= 0.103, *p* < 0.01; Figure 4b).

## 4 DISCUSSION

### 4.1 The chromosome number and karyotype of *Lycoris radiata*

Several cytological studies on *L. radiata* have been made by various authors (Bose, 1963; Kurita, 1987; Nishiyama, 1928; Qin et al., 2004; Shao et al., 1994; Zhou et al., 2007). Recently, Liu et al. (2016) reported the somatic chromosome numbers of four hundred and sixty‐six individuals from 25 populations of *L. radiata* in China, of which 10 were diploid (2*n* = 22) and 15 were triploid (2*n* = 33), and no tetraploid cytotype was found. In this study, 1,224 diploid individuals, 1,195 triploid individuals, and one tetraploid individual were detected. Despite the small proportion for the tetraploid, these new counts, together with data from the previous reports (Kurita, 1987; Liu et al., 2016; Zhou et al., 2007), further confirm that *L*. *radiata* is a species complex concluding diploid with 2*n* = 22, triploid with 2*n* = 33, and tetraploid with 2*n* = 44.

The common karyotype of *L. radiata* only consists of typical acrocentric chromosomes (A‐type chromosome) with different types of SATs combination. To date, many abnormal karyotypes of this complex have been reported, such as 2*n* = 22 = 1*m* + 20*A* + 1*B* (Shao et al., 1994); 2*n* = 33 = 1*m* + 31*A* + 1*B*, 2*n* = 32 = 1*m* + 31*A* (Bose, 1963; Kurita, 1987); 2*n* = 21 = 1*m* + 10*st* + 9*T* + 1*B* (Zhou et al., 2004); 2*n* = 21 = 1*m* + 20*st*, 2*n* = 25 = 1*m* + 20*st* + 2*t* + 2*T* (Zhou et al., 2007), due to the various rearrangements of A‐type chromosome. Through careful examination on the karyotype of 519 bulbs of *L. radiata* in Japan, Kurita (1987) observed 14 bulbs with aberrant chromosomes, whereas no bulb with aberrant chromosomes was found by Liu et al. (2016) based on the cytological study of 466 bulbs of this species in China. As reported in the previous studies, some rearranged chromosomes from A‐type chromosome which are aberrant have been also found in this paper. The number of bulbs with aberrant chromosomes is small, only accounting for 1.32% of total examined bulbs (32/2,420), and the aberrants could be classified into three types and include metacentric chromosome (*m*), small metacentric chromosome (*m*′), and B‐type chromosome. The diploid populations have less abnormal individuals with aberrant chromosomes than the triploid populations. Because the diploids have both sexual and asexual reproduction, and the individual with aberrant chromosomes producing no seeds might be gradually excluded from the population due to intraspecific competition, so less individuals with aberrant chromosomes were found from the diploid population than from the triploid population.

The phenomenon of Robertsonian fusion of A‐type chromosome was found in some bulbs from five different populations. As confirmed in some previously published researches, the m‐type chromosome in one diploid bulb from population 40 may be produced by the fusion of two A‐type chromosomes. Concerning the origin of the triploid individuals carrying aberrant m‐type chromosome, there are two alternative explanations. The one is that the m‐type chromosome was generated from a diploid population including the bulb with m‐type chromosome by hybridization between a normal haploid gamete and an unreduced diploid gamete; the other is that it started from a somatic cell of a bulb having only A‐type chromosomes, in which m‐type chromosome was formed by Robertsonian change, as it was observed in the diploid bulb of population 40. Which explanation is reasonable, hybridization or somatic cell mutation? Eleven abnormal triploid bulbs with m‐type chromosome had been also found by Kurita (1987). He considered that the latter, somatic cell mutation, might be the actual occurrence, because only a very small proportion of examined bulbs carried the aberrant m‐type chromosome. The authors agreed with Kurita’ opinion, considering that the m‐type chromosome in aberrant diploid and triploid bulbs should be produced by Robertsonian fusion.

Although the karyotypes of *L*. *radiata* have some variability among inter‐populations and even within intra‐population to some extent, it was convinced that *L*. *radiata* have a relatively stable karyotype composed of 22/33/44 A‐type chromosomes on the basis of large‐scale sampling from China and Japan.

### 4.2 The distribution patterns of cytotypes and origin of polyploids

Information on the geographical variation of cytotypes is critical for studies of origin and evolution of polyploids (Wu et al., 2016). A detailed investigation of the distribution of diploids and derivative polyploids can provide critical insights into the origins and establishment of new polyploids and cryptic speciation within a morphological species (Baack, 2004; Odee, Wilson, Omondi, Perry, & Cavers, 2015; Segraves, Tompson, Soltis, & Soltis, 1999; Steussy, Weiss‐Schneeweiss, & Keil, 2004). In this study, we conducted an exhaustive survey of the chromosomal races in most populations within the natural range of *L*. *radiata* complex across the entire distribution regions in China.

Although whether or not polyploids have a broader niche breadth than diploids remains controversial (te Beest et al., 2012; Glennon, Ritchie, & Segraves, 2014; Martin & Husband, 2009), in numerous complex species documented by many authors (Lowry & Lester, 2006; McIntyre, 2012; Treieret al., 2009), the polyploids show wider geographic ranges and greater stress tolerances of extreme ecological conditions. In comparison with the diploids, the triploids have a significantly larger geographic range. The triploids distribute from the southwest of China eastward to the south of Korea and south of Japan, covering the whole geographic distribution ranges of the complex. In contrast, the diploids are primarily limited to the middle and east of China, with some diploid populations scattering in the southeast of China. Together with some previous reports (Kurita, 1987; Liu et al., 2016), it can be concluded that the triploid shows a significantly wider distribution range than the diploid, with the former occupying almost the whole distribution range of this complex in East Asia.

A positive correlated relationship between polyploidy and elevation is fairly well supported (Brochmann et al., 2004; Soltis, 1984; Stebbins, 1984). However, in several other cases, the polyploids occupy the lower latitude localities, and a negative correlation of polyploidy with elevation was found, for example, *Atriplex confertifolia* (Stutz & Sanderson, 1983), *Chamerion angustifolium* (Husband & Schemske, 1998), *Centaurea jacea* (Hardy, Vanderhoeven, Loose, & Meerts, 2000), and *Isoetes* spp. (Liu, Gituru, & Wang, 2004). In general, the triploids tend to prefer such place as roadsides, riversides, and the edges of rice paddies or farmlands, and occupy the high altitude regions. Diploids are specific to undisturbed or less‐disturbed habitats, frequently growing under forests, in the lower latitude localities. Moreover, the triploids are expected to present a stronger tolerance to cold temperature, because the triploids show a higher relative distribution dominance than the diploids at higher latitudes. In regard to the north boundary of *L. radiata* distributed in East Asia, the latitude value of diploid and triploid is about 32.6° and 39.0°, respectively. However, this hypothesis needs to be empirically confirmed by further controlled experiments in common garden or greenhouse.

In addition, there is a complicated and perplexing question about the origin and distribution pattern of the polyploids, especially the triploid. The triploid was usually supposed to be an autotriploid (Hayashi, Saito, Mukai, Kurita, & Hori, 2005; Kurita, 1987; Nishiyama, 1928). About the origin of the triploid, there are two key hypotheses. The first is that they are generated from the hybridization of diploid with tetraploid. The second is that they are derived from a crossing between a normal haploid gamete and a nonreduced diploid gamete (Hsu et al., 1994; Kurita, 1987; Zhou et al., 2007). Because the tetraploid has only recently been discovered (Zhou et al., 2007; this paper), most researchers agree to the latter interpretation (Hayashi et al., 2005; Kurita, 1987). To date, only two mixploid populations with the tetraploid cytotype are found, and no independent tetraploid population is detected. In view of the very few tetraploid individuals in natural populations, the authors also agree with the latter explanation.

However, based on an extensive cytogenetical study on the Japanese triploid populations, Kurita (1987) thought that *L*. *radiata* var. *radiata* is not a simple autotriploid. Namely, the triploid is structurally heterozygous at least in regard to the SAT chromosomes (Kurita, 1987). By analyzing the nucleotide sequences of genomic DNA regions in 15 triploid strains and two diploid strains from Japan and China, Hayashi et al. (2005) found some genetic variations between the Japanese and Chinese triploid strains, indicating that *L*. *radiata* var. *radiata* is not a typical autotriploid, supporting Kurita's notion. In our extensive field investigations, we found that the triploids in China also have different SAT chromosome combination, supporting their notion.

Another perplexing problem is why the triploids are distributed very commonly in Japan and South Korea where no diploid mother taxon can be found. The diploids are only distributed in China, and no diploid cytotype has been found so far in Japan and South Korea. Based on the genetic constancy of Japanese triploids in both the nuclear and chloroplast DNA sequences (Hayashi et al., 2005) and the monomorphism on all 24 allozyme loci in Korean *L*. *radiata* populations (Chung, 1999), they thought that the sterile triploids in Japan and South Korea were introduced from China, that is, one and more triploid bulb were brought to Japan and South Korea firstly, and then via extensive asexual reproduction by the rapid formation of new bulbs the triploid spread throughout Japan (except Hokkaido) and South Korea, accompanying human activities to some extent, such as rice cultivation and movement of monks in temples (Chung, 1999; Hayashi et al., 2005; Kurita, 1987). Because the peripheral regions of China, including Yunnan, Guangxi, and South Guizhou possess only triploid populations, as Japan and South Korea do, it is conceivable that it may be the same reason responsible for the formation of the current distribution pattern of *L. radiata* complex cytotypes and nonexistence of the diploid population on the periphery of the distribution range.

With regard to the geographical patterns of different cytotypes of *L. radiata* complex, especially the triploids, there are two possible interpretations. The one is that the triploids are generated from the diploids located in the middle and east of China, then they spread to the surrounding areas where the triploids generally prefer more local environment; the other is that the parental diploid taxon which once had been distributed relatively widely in China, Japan, and South Korea was extinct in Japan, South Korea and many peripheral distribution areas of China for some unknown reasons, only leaving the triploid bulbs. In order to find the key to the questions of the origin, migration routes and distribution patterns of polyploids of *L. radiata*, some molecular and cytogenetic methods are needed in the future study.

## CONFLICT OF INTEREST

None declared.

## AUTHORS' CONTRIBUTIONS

Liu, K. and Zhou, S.B. designed the research. Liu, K. and Meng, W.Q. collected the samples. Liu, K., Wang, L.D., Meng, W.Q., and Zheng, L. generated and analyzed the data. Liu, K. and Zhou, S.B. wrote the manuscript.

## Data Availability

The data supporting the conclusions of this manuscript can be found in the manuscript.
